# Retraction: Dynamic interplay of innate and adaptive immunity during sterile retinal inflammation: insights from the transcriptome

**DOI:** 10.3389/fimmu.2025.1583947

**Published:** 2025-03-06

**Authors:** Frontiers Editorial Office

Following publication, concerns were raised regarding the integrity of the images in the published article. Specifically, immunofluorescence images in Figure 6A and western blot images in Figure 6I were reused in other publications by the same research group. During our investigation, conducted according to Frontiers’ policies, the authors were unable to provide the raw images and a satisfactory explanation. As a result, the data and conclusions of the article have been deemed unreliable, and the article is being retracted.

Christine A. Wells, Aaron Chuah, Hardip Patel, and Elizabeth Mason sent an email to Frontiers requesting the retraction. The authors Krisztina Valter, Matt Rutar, Ricardo Natoli, and Jan Provis agreed to the retraction.

This retraction was approved by the Chief Executive Editor of Frontiers.

